# SlicerHeart: An open-source computing platform for cardiac image analysis and modeling

**DOI:** 10.3389/fcvm.2022.886549

**Published:** 2022-09-06

**Authors:** Andras Lasso, Christian Herz, Hannah Nam, Alana Cianciulli, Steve Pieper, Simon Drouin, Csaba Pinter, Samuelle St-Onge, Chad Vigil, Stephen Ching, Kyle Sunderland, Gabor Fichtinger, Ron Kikinis, Matthew A. Jolley

**Affiliations:** ^1^Laboratory for Percutaneous Surgery, School of Computing, Queen's University, Kingston, ON, Canada; ^2^Department of Anesthesiology and Critical Care Medicine, Children's Hospital of Philadelphia, Philadelphia, PA, United States; ^3^Isomics, Inc., Boston, MA, United States; ^4^Software and Information Technology Engineering, École de Technologie Supérieure, Montreal, QC, Canada; ^5^Pixel Medical, Kingston, ON, Canada; ^6^Department of Radiology, Brigham and Women's Hospital, Harvard Medical School, Boston, MA, United States; ^7^Division of Cardiology, Children's Hospital of Philadelphia, Philadelphia, PA, United States

**Keywords:** computer modeling (simulation), pediatric cardiology and surgery, 3D echocardiography (3DE), open-source, image-based modeling, cardiac valves

## Abstract

Cardiovascular disease is a significant cause of morbidity and mortality in the developed world. 3D imaging of the heart's structure is critical to the understanding and treatment of cardiovascular disease. However, open-source tools for image analysis of cardiac images, particularly 3D echocardiographic (3DE) data, are limited. We describe the rationale, development, implementation, and application of SlicerHeart, a cardiac-focused toolkit for image analysis built upon 3D Slicer, an open-source image computing platform. We designed and implemented multiple Python scripted modules within 3D Slicer to import, register, and view 3DE data, including new code to volume render and crop 3DE. In addition, we developed dedicated workflows for the modeling and quantitative analysis of multi-modality image-derived heart models, including heart valves. Finally, we created and integrated new functionality to facilitate the planning of cardiac interventions and surgery. We demonstrate application of SlicerHeart to a diverse range of cardiovascular modeling and simulation including volume rendering of 3DE images, mitral valve modeling, transcatheter device modeling, and planning of complex surgical intervention such as cardiac baffle creation. SlicerHeart is an evolving open-source image processing platform based on 3D Slicer initiated to support the investigation and treatment of congenital heart disease. The technology in SlicerHeart provides a robust foundation for 3D image-based investigation in cardiovascular medicine.

## Introduction

Cardiovascular disease is a significant cause of morbidity and mortality in both children and adults. Patient-specific analysis and treatment of cardiovascular disease is increasingly dependent upon multimodality 3-dimensional (3D) imaging such as 3D echocardiography (3DE), cardiac magnetic resonance imaging (CMR), and computed tomography (CT) (1–5). Applications for viewing and analyzing 3D cardiovascular images, and in particular, 3DE images, are primarily commercially developed systems targeting specific high-incidence applications in adult cardiovascular medicine. Further, most currently available commercial cardiac image visualization and analysis programs cannot be significantly customized or extended by the end user to meet the needs of small or unique populations.

Patient-specific, image-derived modeling and simulation are particularly relevant to the treatment of congenital heart disease (6, 7). A highly customized approach to surgical repair and intervention is frequently needed due to the inherently atypical and variable anatomy (8). However, congenital heart disease is unlikely to elicit significant development of dedicated modeling software by industry as it consists of small and heterogeneous populations (9). Similarly, collaborative academic efforts spanning multiple institutions (e.g., The Pediatric Heart Network) are often needed to assemble sufficient sample sizes for adequately powered studies (10). While the needs for customization, innovation, and readily shared tools are particularly critical to the advancement of image-derived modeling for this vulnerable population, these tenets remain relevant in the broader context of cardiovascular science. As such, continuous advances in multi-modality 3D imaging and image processing create the opportunity for the complementary development of open software tools for the visualization, modeling, quantification, and treatment planning in cardiovascular disease, particularly for “technologically orphaned” populations with congenital heart disease.

The initial impetus for this work was our desire to investigate the structure of atrioventricular heart valves in children with congenital heart disease using 3DE images. We could not find an existing image analysis platform that would allow 3DE import, viewing, volume rendering, and custom modeling with sufficient flexibility to perform this investigation. While there were existing projects with specific foci that partially met our needs, none provided all of the features for 3DE-derived cardiac modeling that we envisioned (11–13). Therefore, we developed and implemented SlicerHeart, our open-source extension for 3D Slicer. 3D Slicer is a free, extensible, and open-source software application for medical image computing. We selected the 3D Slicer platform as our base because it has a large international community of both users and developers with over 10,000 downloads per month, and existing, well-established mechanisms for adding functionality (14–16). Notably, the licenses for both SlicerHeart and 3D Slicer are specifically unrestrictive BSD-like licenses, allowing incorporation of algorithms and components freely into academic work and commercial applications.

3D Slicer includes tools for data import, visualization, image analysis, interaction, surgical navigation, radiomics, and even machine learning (14–20). However, until SlicerHeart, 3D Slicer did not have the ability to import or visualize 3DE data, volume render 3DE data, nor have the workflows dedicated to the evaluation and modeling of cardiovascular structures such as cardiac valves. Although the initial development of these tools was focused on pediatrics and 3DE images, we have now demonstrated application to multiple cardiovascular diseases with clear relevance to all ages (7, 21–29). Despite not being formally released until now, at the time of writing, SlicerHeart has been downloaded over 43,000 times since inception in 2015. As such, we hope the SlicerHeart extension will form the basis for future investigations aimed at unveiling the wealth of latent information in 3D cardiovascular images and be a catalyst for transparent and reproducible science. The remainder of this manuscript focuses on the rationale for and dedicated development of tools for the analysis of 3D cardiac imaging, and specific applications, with an emphasis on 3DE and application to congenital heart disease.

### Part I. Rationale for and development of SlicerHeart platform

3D Slicer's application framework provides all the essential features of a general medical image visualization and analysis application, such as standard Digital Imaging and Communications in Medicine (DICOM) import and export, 2D image slice and 3D visualization, image segmentation, registration, quantification, time sequences, plots, and annotations (16). It also includes many advanced features including artificial intelligence-based processing, the capability for immersive stereo virtual reality visualization, and real-time connection to hardware devices (e.g., surgical navigation systems) (14, 15, 20, 27). 3D Slicer is also fully extensible using Python scripting. However, there are still specific functions that are necessary to apply this vast array of tools to cardiac data, and particularly 3DE data. The following describes the challenges of integrating cardiac data and the functional solutions we created to address these challenges within SlicerHeart. This study was approved by the Institutional Review board at the Children's Hospital of Philadelphia under IRB protocols 16-013091, 16-012853, and 17-014241.

#### Data import

Unlike CT, MRI, and even 2D echocardiographic images, there is, to date, no widely used standard file format for 3D echocardiographic data. The current DICOM standard does specify an “Enhanced US Volume” information object for 3DE data storage, but all device manufacturers that we have encountered so far utilize their own mutually incompatible and proprietary formats. There are valid reasons why vendors delay adoption of a standard (devices are still in experimental stage, immature standards, standard solution would require more computational or storage resources, etc.). However, the lack of maturity of a standard is a clear roadblock to inter-vendor compatibility of commercial applications as well as independent research. Raw signal data acquired by ultrasound machines, such as data right after beamforming, or before compression, or scan conversion, may be very large, complex, difficult to store in a standard format, and hard to interpret for third-party software. However, these problems are the same for most other imaging modalities (e.g., CT, CMR, Positron Emission Tomography) and the solution has been to reconstruct a regular orthogonal (“Cartesian”) volume from raw data and save that in standard DICOM format. This approach has been adopted by Philips Medical (Andover, MA). Conversion to a Cartesian format requires their Qlab software (Philips Medical, Andover MA), which contains a Cartesian volume export feature (Supplementary Video 1). However, this Cartesian format does not contain associated color Doppler or EKG information. At time of writing, the export format does not completely follow the DICOM standard and therefore most general-purpose DICOM readers, including the basic importer in 3D Slicer fail to import these images.

To address this in the Philips 3DE that we primarily use at our institution clinically, we developed a software module (Philips4DUSDicomPatcher) in SlicerHeart which repairs missing DICOM tags, allowing import of 3DE into 3D Slicer and other standard image processing toolkits (Supplementary Video 1). However, this does not solve the larger challenge of importing data from multiple vendors, and EKG information and 3D color are not available *via* this method.

There has been a recent effort to develop further inter-compatibility across vendors named Image3dAPI, an application programming interface (API) for inter-vendor exchange of 3D ultrasound data (https://github.com/MedicalUltrasound/Image3dAPI). Image3DAPI can be used to read 3D ultrasound images from GE, Canon, Hitachi, Siemens, and Philips's scanners. In addition to the API itself, a library must be obtained from the scanner's manufacturer and installed on the system. Multiple vendors will now provide their commercial library. To allow import of data made available by the combination of Image3dAPI and the vendor provided library we have made the.3dus format output of 3DAPI drag-and-drop compatible with SlicerHeart and 3D Slicer. To demonstrate this functionality, we used the GE library which is readily available from GE, and Image3dAPI to import GE 3DE into 3DSlicer for our validation study (Appendix). As such, the combination of 3D Slicer and Image3dAPI support a wide variety of 3DE vendor formats.

While 3DE provides real-time imaging with unparalleled frame rates and details, other imaging modalities, such as CT and CMR have other unique advantages, such as larger field of view, better distinction of tissues, or functional information. These modalities are supported by the existing import utilities in 3D Slicer core functionality based upon the Common Toolkit (www.commontk.org), or implementations subsequently described as part of the development of SlicerHeart.

#### Cine viewing of 3D volumes

3DE data is typically viewed by serially displaying individual volumes of data over time. As such, after data was imported the next challenge was to play and control sequential 3DE volumes. We created the Sequences module to address this which has now been integrated into 3D Slicer core program. Sequences allows the creation and visualization of higher-dimensional data such as cine or 4D volumes (3D + time) such as 3DE, cine CT, and CMR, using familiar icons and controls.

#### Standard orientation of 3D echocardiographic data

Unlike CT and CMR data, there is no defined standard anatomic orientation for echo images that is registered to an external coordinate system. There are multiple probe orientations for 3D echocardiography on the chest (transthoracic echocardiography, TTE) and in the esophagus (transesophageal echocardiography, TEE). Further, even with “standard views” the position on the chest and exact angle relative to the body and heart may vary. Echo images are acquired relative to the position of the heart and are somewhat independent of the position of the probe on the body. For example, in dextrocardia, the apical position is on the right side of the chest, as opposed to the left chest in levocardia. In pediatrics, 3D TTE imaging is even more critical as there is no currently available 3D TEE probe suitable for small children. Images are routinely acquired from apical, subcostal, and parasternal views. As such, we required a systematic means of orienting and displaying both 2D and 3D on screen views of the echo data from multiple acquisition positions, as well as means of reorienting the data to the desired viewpoint for a particular task. We could not find a convention that was accurately implemented in commercial software to guide us. As such, first, we created presets which oriented the image anatomically relative to a heart model avatar for different imaging positions (apical 4-chamber, subcostal, mid-esophageal) as well as the “patient anatomical axis” used as convention in CT and MRI. The avatar heart model remains registered to the echo images after transform to help maintain orientation for the user.

#### 2D cross-sectional visualization of 3D imaging including 3DE

The desired image analysis software needed to be able to display imported images in various representations. Cross-sectional image slice visualization of volumetric images is commonplace, and methods that are used for other imaging modalities, such as CT and CMR are applicable to 3DE images. Clinical procedures that involve placement of implants or require visualization of 3D computational models may require display of this information in off axis cross-sectional views. For example, for valve analysis it may be preferable to set orientation based on view orientation preset (as described in previous section) and rotate linked orthogonal views around an axis orthogonal to the third slice view (such as the plane of the valve annulus). We implemented such viewing planes which can be utilized in 3DE, CT and CMR images.

#### Volume rendering 3DE and tomographic images

Direct volume rendering is a technique used in many medical imaging applications including the clinical display of 3DE data. Notably, unlike segmentation, volume rendering can be nearly instant on modern hardware and as such is a practical way to display cine volumes (3D + time). Volume ray casting is a commonly used direct volume rendering method, allowing to accumulate information on voxel intensity along a casted ray through a 3D dataset (see Appendix). To meet specific needs for the rendering of cardiac 3DE, we have created the Echo Volume Render module within SlicerHeart (Supplementary Video 2). The module builds upon basic functionalities within the 3D Slicer Volume Rendering module and the underlying Visualization Toolkit (VTK) (18), but has been significantly extended and customized for the volume rendering of 3DE. For example, we have integrated commonly used features such as depth coloring, smoothing, thresholding, and designation of a region of interest, analogous to features on clinically utilized 3DE platforms.

A commonly used technique in volume rendering of 3DE is depth-dependent coloring, for which coloring is based on the voxel's distance from the observer, instead of being determined by the voxel's intensity value (see Appendix). Commercial platforms often use a yellow-to-blue hue convention, with blue being further into the background. We added this coloring method by creating a custom depth-dependent shader for the module. Coloring dependent on the orientation of the intensity gradient has also been added to help with depth perception (see Appendix).

##### Smoothing factor

**Figure 1:**
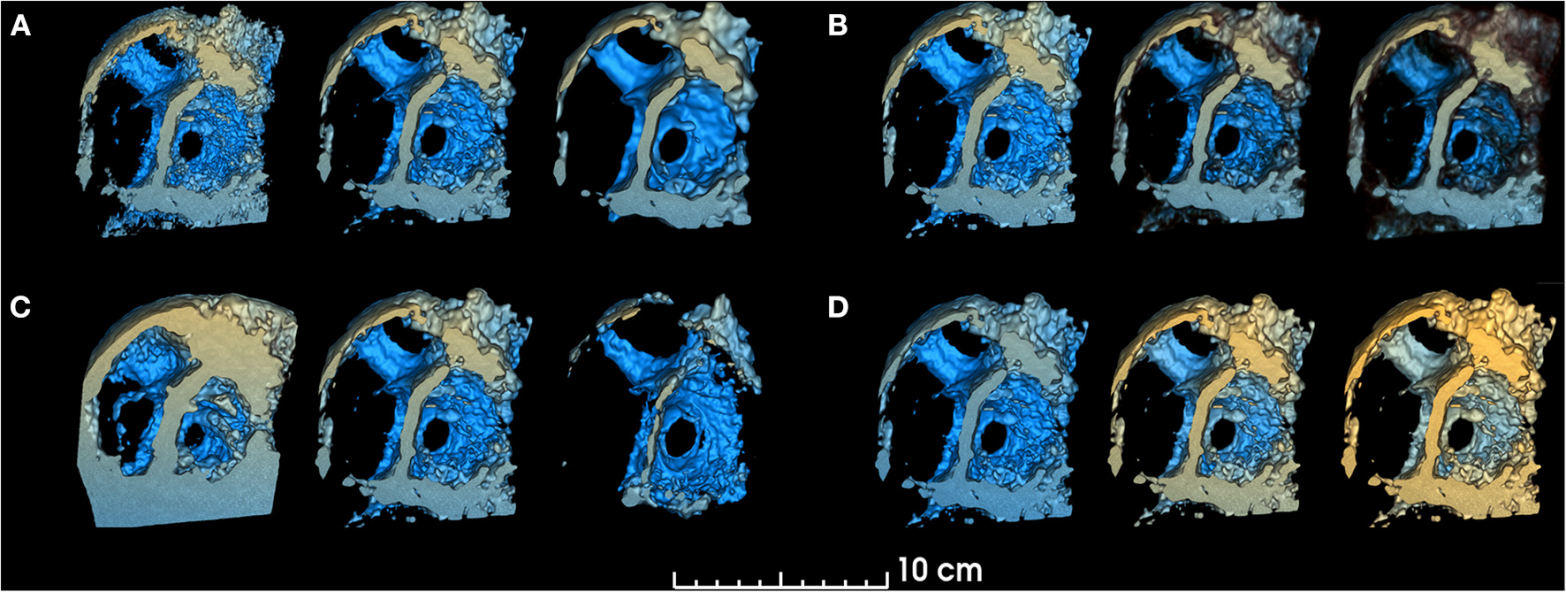
Smoothing, threshold, edge smoothing and depth range parameters. changes in volume rendered model for different values of **(A)**. Smoothing (left: 0.25, center: 1.0, right: 1.75), **(B)** Threshold (left: 30%, center: 50%, right: 70%), **(C)** Edge smoothing (left: 0%, center: 10%, right: 20%), **(D)** Depth range [left: (−150, +10), center: (−103, +40), right: (−103, +80)]. Unchanged parameters have remained constant at the following value: Smoothing factor = 1.00, Threshold = 50.9%, Edge smoothing = 2.0%, Depth range = [−103, 10], Depth darkening = 30.0%, Depth coloring = [−24.35, 23.55%], Brightness = 165%, Saturation = 200%.

Speckle noise is a distinct characteristic of 3DE that interferes with anatomical interpretation by decreasing the ability to identify details and edges (30). To address this issue, a “smoothing factor” parameter has been implemented to apply a Gaussian smoothing filter to the 3D dataset, which computes the average intensity of neighboring voxels and therefore “blurs” the volume to reduce noise in 3DE images prior to volume rendering (Figure 1).

##### Threshold and edge smoothing

**Figure 2:**
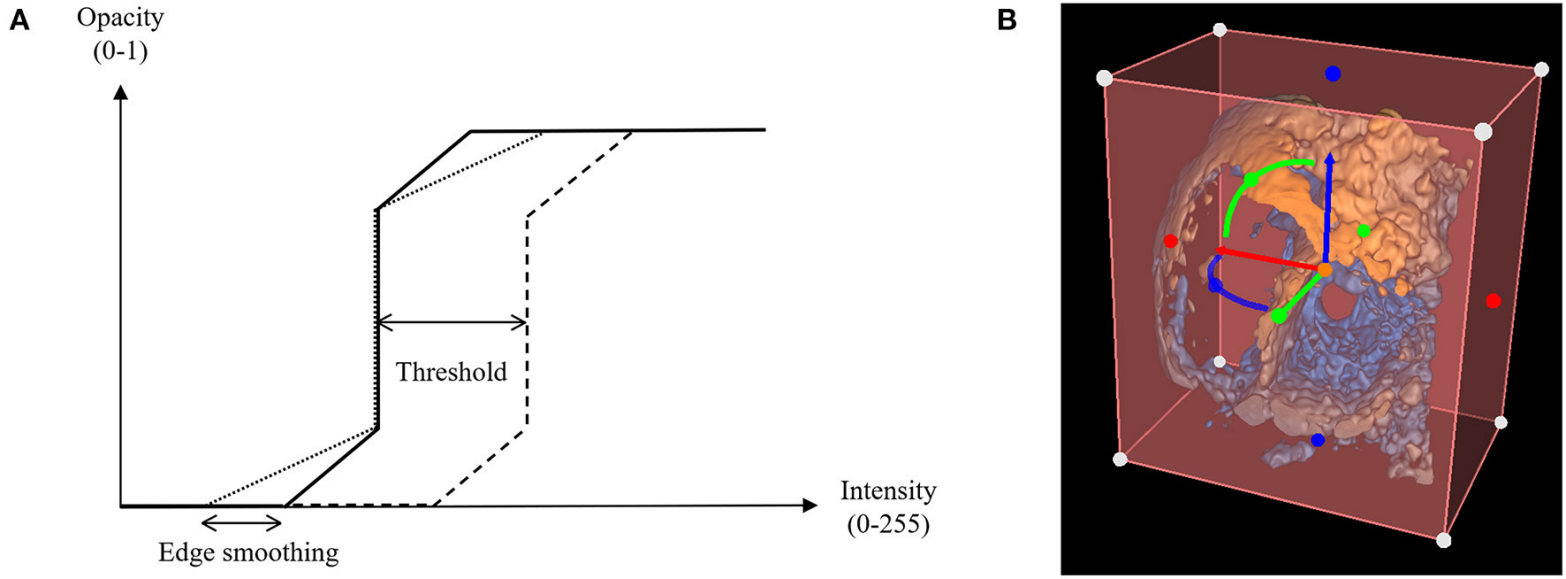
Threshold and edge smoothing parameters for changes in opacity. **(A)** Opacity is shown as a function of voxel intensity, where voxels having an intensity value greater than the upper limit appear completely opaque and voxels with an intensity value inferior to the lower limit appear invisible, having a zero-opacity value (see Appendix); **(B)** Selection of a Region of Interest (ROI) to enable cropping of the rendered 3D echocardiographic volume.

The standard volume rendering module of VTK maps voxel intensity to opacity values using what is commonly called a transfer function. To easily manipulate this function, two simple parameters have been added: threshold and edge smoothing (Figures 1, 2).

##### Depth range, depth darkening and depth coloring

The “depth range” parameter allows the user to modify the range for depth-dependent coloring, by changing the range of yellow and blue hues (Figure 1). The “depth darkening” parameter allows the darkening of more distant structures to help with depth perception. To our knowledge, while the yellow-to-blue hue is the convention used by commercial platforms, no readily available study has been conducted to determine if these are the optimal colors for viewing 3DE. As such, a “depth coloring” functionality has been added to modify the yellow-to-blue hue to different colors (Supplementary Video 2).

##### ROI and cropping

The creation of a region of interest (ROI) enables cropping of the volume rendered image to clearly visualize specific anatomical structures. Cropping often needs to be performed off axis from the initial image, requiring the cropping object to be transformed. We integrated intuitive cropping and transformation into the Echo Volume Render Module, which allows cropping and rotational alignment in 2D and 3D views based on widget functionality we recently developed within 3D Slicer (Figure 2 and Supplementary Video 2).

#### Image segmentation

Segmentation of medical images is the process of assigning a value to a given pixel (in 2D) or voxel (in 3D). The goal of segmentation is to simplify the representation of an image to make it intuitive to visualize and analyze. For example, segmentation is an essential part of the workflow in the conversion of images to models suitable for 3D printing. Segmentation can be performed on any data, including 3DE using the tools we have released, using the Segment Editor and Segment Editor Extra Effects modules we have created (31). Customized workflows can then be created for dedicated processes such as the segmentation and modeling of specific structures (32, 33). For example, segmentation of heart valves can be difficult, and benefits from dedicated workflows to procedurally align images for optimal identification of the valve annulus and leaflets. The following describes implementations which inform model creation and analysis within dedicated modules in SlicerHeart.

#### Cardiac valve annular modeling and quantitative analysis

**Figure 3:**
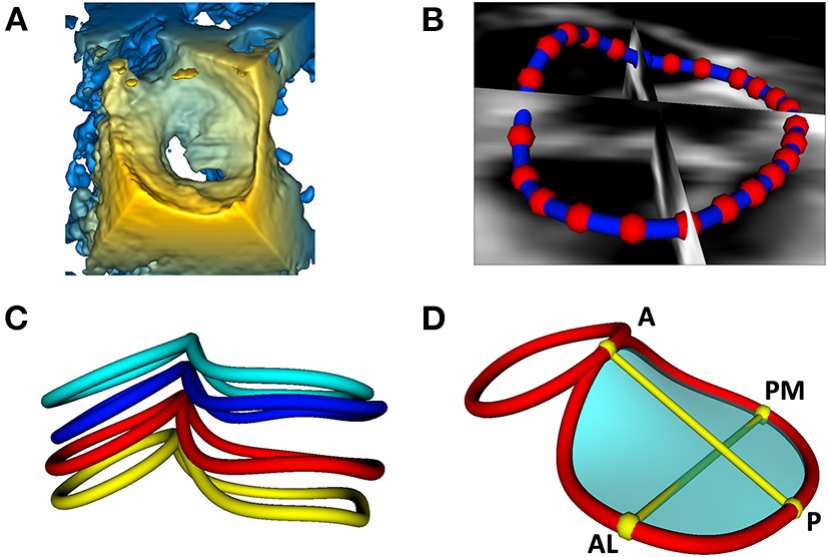
Visualization and modeling of mitral valve annuli. **(A)** Volume rendering of a 3DE of a mitral valve using the echo volume render module in SlicerHeart. **(B)** Curve creation of annular model of a mitral valve visualized through 2D slice intersections using valve annulus analysis module in SlicerHeart. **(C)** Visualization of mitral and aortic annular curves in all four phases of the cardiac cycle (top to bottom: End Diastole, Mid Diastole, Mid Systole, End Systole). **(D)** Quantification of mitral and aortic annuli using valve quantification module in SlicerHeart. 3DE, 3D echocardiogram; A, Anterior; P, Posterior; AL, Anterolateral; PM, posterior medial.

The initial driving rationale and motivation for the above capabilities was to allow the modeling of the structure of pediatric heart valves using 3DE. An important component of valve function is the fibrous support structure around the valve known as the annulus. The mitral annulus has been the focus of substantial 3D image-based research resulting in a paradigm shift in the way both mitral valve assessment and surgical repair are performed (34, 35). There are several closed-source proprietary tools for mitral annular modeling, but none allow for user customization, or further modification such as building modeling and quantification for other valve types (e.g., complete atrioventricular canal) (3, 36–38). Further, the methods in commercial platforms are not reproducible by those without those programs, or transparent regarding the underlying methods utilized. As such, we have created a custom workflow for the evaluation of the valve annulus which allows alignment of the views to the plane of the annulus, placement of paired points by rotating around an axis orthogonal to the annular plane, and then annular smoothing using Fourier smoothing (Supplementary Video 3) (39). Points of interest for the mitral annulus include the anterior (high point adjacent to the aortic annulus), posterior, antero-lateral, and posterior-medial points (Figure 3). Once these points are placed, the annular area, circumference and respective diameters are automatically calculated and visualized. Other commonly used metrics are automatically generated (Supplementary Video 4). However, an infinite number of scenarios can be created using basic Euclidian metric, as we have demonstrated for the tricuspid valve and complete atrioventricular canal valve (26, 40). Notably the module is agnostic to data type, and can be applied to 3DE, CT, rotational angiography, CMR or any other type of data which can be imported into 3D Slicer (7).

#### Leaflet modeling and quantitative analysis

The majority of commercial valve leaflet modeling software released to date define valve model as infinitely thin manifold surfaces approximating the surface of the leaflets. Volumetric modeling (based on segmentation) allows application of image analysis tools which have been created for volumetric modeling, and recently customized to valve modeling (41, 42). Segmentation-based modeling has benefits relative to thin surface models: (1) It captures all the available information of the structure in the image (2) it allows the utilization of robust image processing techniques (3) each segmentation can serve as a potential atlas for automatic segmentation techniques (such as machine learning) (28, 43, 44). Of course, it also allows creation of stereolithography (.stl) files suitable for 3D printing and 3D printing-based simulation (33, 45, 46).

We have previously demonstrated application of the 3D Slicer Segment Editor module and have now incorporated a valve specific segmentation workflow that begins with the assignment of annular landmarks and proceeds to facilitate optimal views for segmentation (31, 33). Leaflets can now be segmented in a dedicated workflow informed by the annular ROI using the assortment of semi-automatic tools available in 3D Slicer including the Segment Editor and Segment Editor Extra Effects modules. Leaflets can then be smoothed by applying various filters, with the default being a median filter with customization we implemented to enforce the critical boundary between leaflets where they coapt (Supplementary Video 3).

Previous work has demonstrated associations of valve leaflet structure to valve function (3, 47, 48). As such we created the Valve Quantification Module (Supplementary Video 4). Mitral leaflet area, tenting height, tenting volume, billow height, and billow volume are automatically calculated in a user-initialized workflow for the valve and individual leaflets. In addition, the workflow allows assessment of valve leaflet coaptation height, length, and area in a novel workflow (Supplementary Video 4).

#### Batch processing and export

Image analysis can generate a large amount of output data which will need to be further analyzed. For example, after generating quantification on a group of valve annuli a user may want to output those quantities (circumference, diameter, area etc.) in a table for statistical analysis. To meet this need we created the Tables module in 3D Slicer to allow the creation, display and editing of spreadsheets generated from data within 3D Slicer. We then created customized batch processing with SlicerHeart to facilitate the analysis of multiple saved valve analysis scenes and allow the output of all metrics generated from those scenes in standardized formats (e.g., csv) for statistical analysis.

#### Dissemination

3D Slicer is available at www.slicer.org. SlicerHeart is available as a pre-built extension with the 3D Slicer Extension Manager with the underlying open-source code available at https://github.com/SlicerHeart/SlicerHeart. 3D example data is available in the same repository and in the Sample Data module in 3D Slicer.

### Part II: Research application of SlicerHeart

#### Modeling mitral valve annuli

We have previously demonstrated application of the Annular Modeling module for a mitral valve using 3DE and cardiac CT (23). Modeling begins by choosing the probe orientation to register the image, then refining the views to align one plane through the plane of the annulus and the other two views orthogonal to the annulus (Figure 3 and Supplementary Video 3). The annular curve can then be defined and quantified as we have demonstrated in application to valves of patients with congenital heart disease (Figure 3, Supplementary Videos 3, 4) (22, 26, 49).

#### Modeling mitral valve leaflets

**Figure 4:**
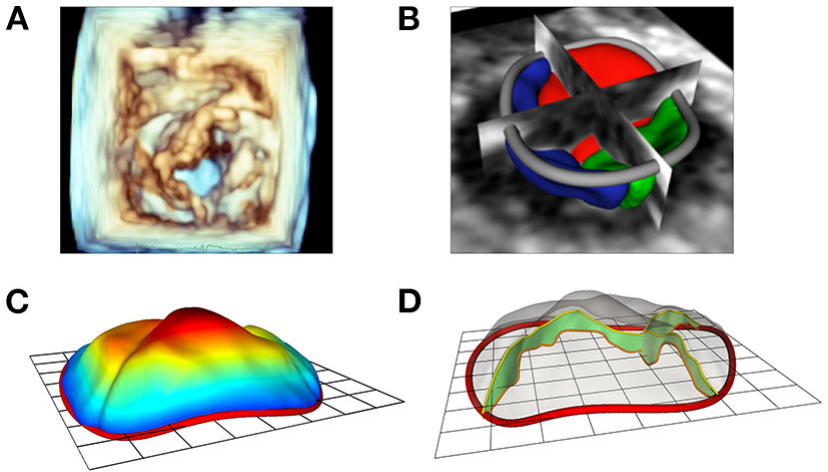
Modeling of valve leaflets and quantification of annular and leaflet structure. **(A)** Volume rendering of a tricuspid valve using Phillips Qlab Software; **(B)** Visualization of a tricuspid valve (right atrial view) segmentation with slice intersections (blue = septal leaflet, red = anterior leaflet, green = posterior leaflet) in SlicerHeart; **(C)** Color intensity map depicting quantitative degree of billow in a tricuspid valve with 2D annular plane visible in SlicerHeart; **(D)** Visualization of leaflet coaptation regions with 2D annular plane visible in SlicerHeart.

The new Leaflet Segmentation module within SlicerHeart builds upon the annular model created in the Annular Modeling module. This customized module leverages the underlying functionality of the Segment Editor module in Slicer with newly customized features to facilitate valve segmentation from 3D images. The Leaflet Modeling module employs the annular model to create a region of interest to mask the valve for application of a multitude of segmentation tools within the Segment Editor and Segment Editor Extra Effects modules (Supplementary Video 3). These models can then be visualized on screen (Figure 4), or exported, including in stereolithography (.stl) format for 3D printing and simulation as we have previously demonstrated (33).

#### Quantification of annular and leaflet structure

**Figure 5:**
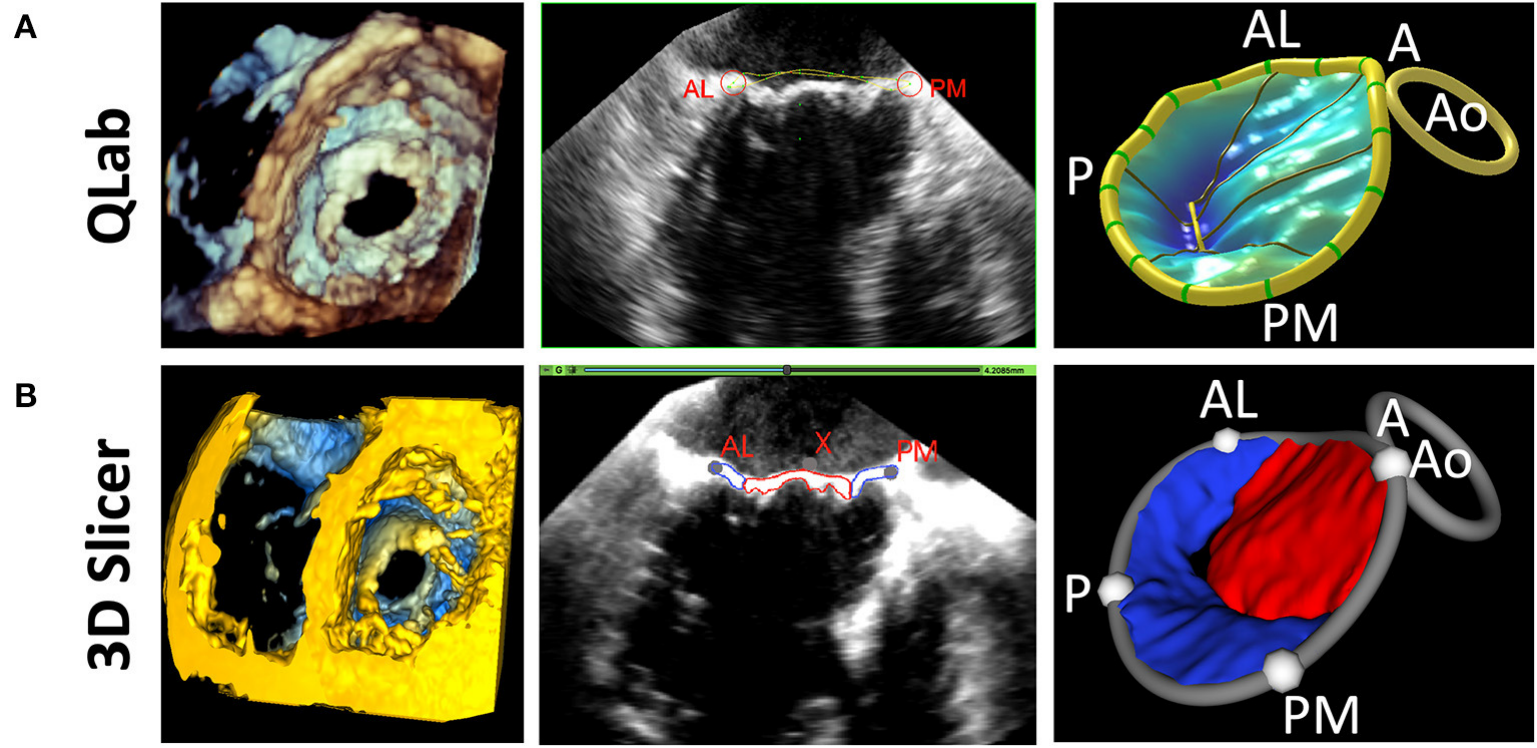
Comparison of 3D Slicer vs. QLab Mitral Valve Quantification (MVN). (Left) volume rendering of a mitral valve viewed from the left ventricle in Qlab **(A)** and 3D Slicer **(B)**; 2D Transesophageal view of the mitral valve highlighting the AL and PM points in Qlab **(A)** and 3D Slicer **(B)**; 3D Model generated in Qlab **(A)** and 3D Slicer **(B)**. Qlab, Philips Medical, Andover, MA.

The new Valve Quantification module within SlicerHeart allows semi-automatic quantification of important annular and leaflet metrics (Figures 4, 5 and Supplementary Video 4). A preliminary version of this workflow has been used to generate mitral annular quantification for assessment of suitability for transcatheter mitral valve as we have recently described (23). Custom adaptations of this workflow have been applied to investigations of the tricuspid annulus in hypoplastic left heart syndrome and of the annulus of the native, unrepaired valve in complete atrioventricular canal (26, 49).

#### Volume rendering of 3D echocardiographic and tomographic data

**Figure 6:**
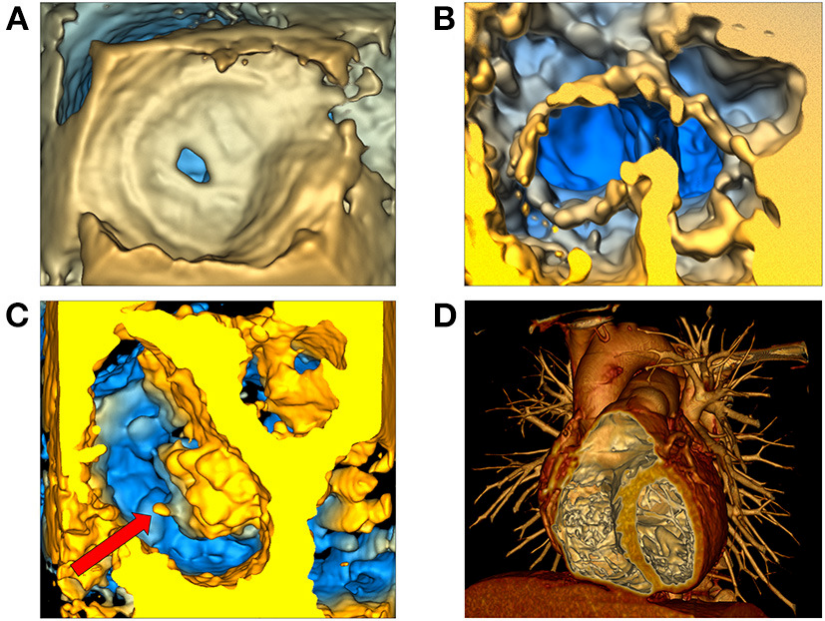
Volume rendering of 3D echocardiographic and tomographic data. **(A)** Visualization of an atrial septal defect in the echo volume render module in SlicerHeart from a right atrial view; **(B)** Visualization of a complete common atrioventricular canal valve in diastole (ventricular view in SlicerHeart); **(C)** Left atrial view of an aortic valve and a mitral valve with anterior leaflet prolapse and a ruptured chord which is visible as indicated by the red arrow; **(D)** CT image of a normal anatomic heart cropped anteriorly to visualize the right atrium and ventricle and the left ventricle using volume rendering in 3D Slicer. CT, Computed Tomography.

The new Echo Volume Rendering module in SlicerHeart incorporates our recent development of 3DE-focused volume rendering functionality, robust image cropping, and user controls in an accessible framework as demonstrated in Supplementary Video 2. The module is designed to be simple and familiar enough for clinical users, while offering substantial control over customization. It also provides all the fundamental framework necessary to catalyze further development of novel rendering algorithms by the research community. The module allows for the simple and rapid visualization of structures such as septal defects, valve leaflets, and the vasculature of patients from 3DE (Figure 6). Finally, given that volume rendering is the primary modality by which 3DE is displayed clinically, it forms the visualization foundation for the introduction of a multitude of modeling and simulation workflows such as transcatheter valve and transcatheter edge-to-edge therapy modeling within volume rendered images as described below.

#### 3D printing and visualization technologies

**Figure 7:**
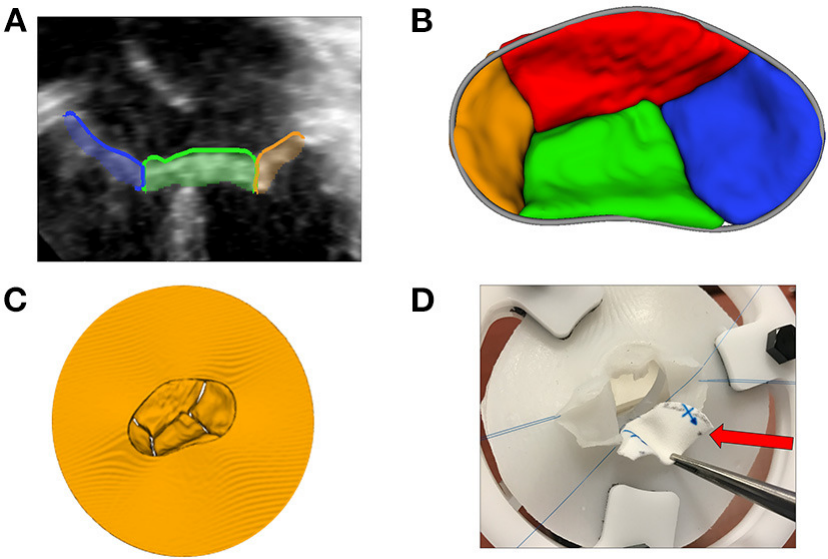
3D printing and novel visualization technologies. **(A)** A 2D transthoracic apical view of a CAVC valve (from image left to right: right mural leaflet in blue, inferior bridging leaflet in green, and left mural leaflet in orange) segmented using the Valve Segmentation module in SlicerHeart; **(B)** An 3D atrial view of a segmented CAVC valve with the annular curve visible in gray; **(C)** A virtual model of the segmented CAVC valve with a skirt used as a template for 3D printing; **(D)** A surgeon practices a patch repair on a AVC valve created from a 3D echocardiogram with the dividing ventricular septal defect patch indicated by the red arrow. CAVC, complete common atrioventricular canal.

3D printing has been described as a transformative technology for congenital heart disease and other cardiovascular applications (6). Notably all models created in SlicerHeart can be exported to stereolithography (.stl) format to enable 3D printing using the export function in the Segmentations module in 3D Slicer. We have demonstrated application of 3D printed simulation of valve modeling including the direct 3D printing of valve models and the creation of molds from the models as shown in Figure 7 (33).

Virtual reality (VR) is an emerging technology that uses a head-mounted display to show immersive view with wide field of view, binocular stereo visualization and allows natural interaction with the displayed content using head, eye gaze, and hand motion tracking. Virtual reality has immense potential to transform the visualization of cardiac 3D data (50). We developed and implemented the integration and volume rendering of cardiac images into SlicerVR (25, 27). The SlicerVR module provides a direct means of visualizing any scene displayed in SlicerHeart in virtual reality, which we have now demonstrated within numerous cardiac-focused workflows (22, 25, 27, 29, 40).

#### Image-based modeling of transcatheter devices

**Figure 8:**
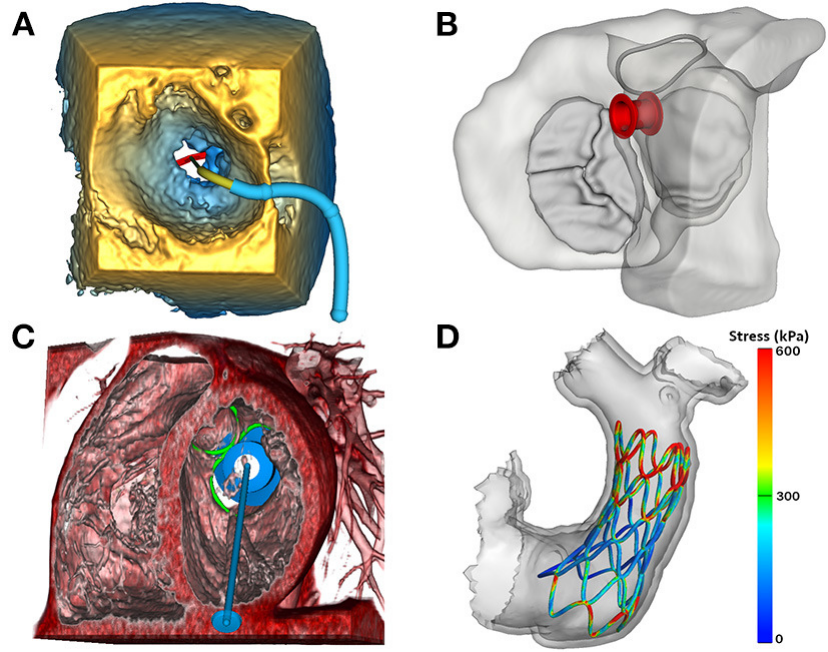
Image-based modeling of transcatheter devices. **(A)** Demonstration of the ValveClip delivery simulator module in SlicerHeart, to model a transcatheter edge-to-edge repair within a 3DE image of a mitral valve rendered in the echo volume rendering module; **(B)** Visualization of an ASD closure device in segmented myocardium and valves viewed from the atrium using the ASD/VSD Device simulator module; **(C)** An apical tether transcatheter mitral valve device positioned in a mitral valve in a CT volume rendering using the transcatheter atrioventricular valve simulator module in SlicerHeart; **(D)** Visualization of a finite element model of a self-expanding valve deployment simulation in SlicerHeart. 3DE, 3D Echocardiography; ASD, Atrial septal defect; VSD, Ventricular septal defect; CT, Computed Tomography.

**Figure 9:**
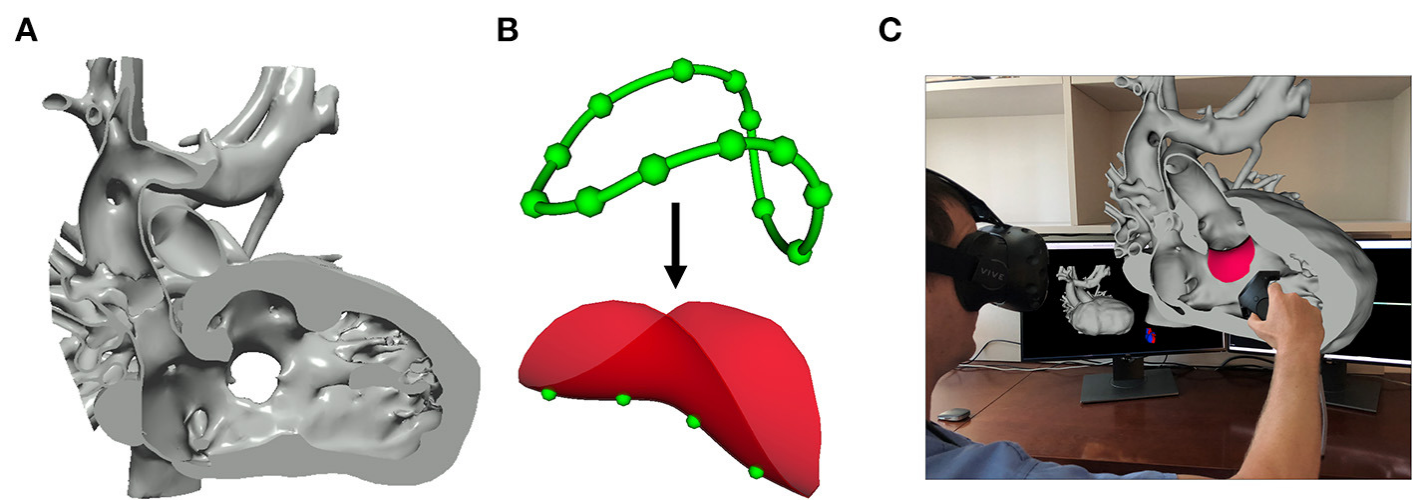
Image-based planning of surgical procedures. **(A)** 3D virtual model of a heart with double outlet right ventricle anatomy cut to visualize the Ventricular Septal Defect that will be baffled to allow for continuous blood flow; **(B)** Closed baffle perimeter contour created by placing points in the 3D heart model which becomes a baffle model onto which surface points may be placed to customize the shape of the baffle; **(C)** Rendering demonstration visualization of the baffle model placed within the 3D heart model in SlicerHeart using the SlicerVR module.

One of 3D Slicer's areas of emphasis is to enable and inform image-guided therapies across multiple domains (20). Transcatheter cardiac interventions are becoming more complex, and image-based visualization and modeling has become an essential part of preoperative planning. We have recently demonstrated utilization of a new functionality within SlicerHeart called the Cardiac Device Simulator Module which we employed to model virtual placement of an evolving group of cardiac devices (Figure 8). We have customized workflows for a diverse group of transcatheter procedures including self-expanding transcatheter pulmonary valves in CT datasets and transcatheter atrial and ventricular septal occlusion devices within 3DE datasets (Figure 8) (22, 32, 40). Further specialization of this general toolkit has now been implemented to support the modeling of transcatheter mitral valves and transcatheter edge-to-edge therapies which we have recently demonstrated (Figure 9) (7, 23). All simulated devices available in the Cardiac Device Simulator Module can be inserted into segmented or volume rendered images (3DE, CT, CMR) and visualized on screen or in virtual reality.

#### Image-based planning of surgical procedures

Segmentation-based modeling and 3D printing has been demonstrated to inform the planning of complex surgical procedures. However, until recently there were not readily available tools focused on the unique challenges in the surgical repair of congenital heart disease. We implemented new functionality to optimally visualize the necessary anatomy in the form of the Dynamic Modeling tool (Figure 9A). The Baffle Planner Module allows for the interactive and simple placement of “surgical” patches or baffles into segmented virtual models of patients with double outlet right ventricle as shown in Figure 9B (29). This workflow is applied clinically at our institution where we have found it to be faster and more intuitive than traditional segmentation-based baffle creation. As such, this application is now utilized to present potential surgical options during weekly preoperative cardiac surgical conferences. We feel this demonstrates the potential power of the integration of tools traditionally associated with computer-aided design (CAD) into a medical image visualization platform such as SlicerHeart, and we are continuing to develop and implement additional features for this purpose. This workflow further demonstrates the flexibility of an open-source, customizable image processing framework to inform image derived planning in patient populations too small and heterogeneous to attract commercial investment.

### Part III: Validation

#### Validation of data representation

The import of tomographic images in DICOM format into 3D Slicer has been previously validated (16). Accuracy of the SlicerHeart 3DE import functionality has been previously demonstrated (33), but not rigorously validated in comparison to commercial platforms on a calibrated ultrasound phantom. Measurements of data in 3D Slicer derived from Philips 3DE data and GE 3DE data converted using the Image3dAPI were compared to measurements performed on the same images of the calibrated ultrasound phantom using commercial software. In comparing measurements between Philips QLab and 3D Slicer, the mean difference is 0.04 mm per 5 mm (0.8% difference) with a maximum error of 0.1 mm per 5 mm (2% difference). In comparing measurements between GE Q Analysis and 3D Slicer, the mean difference is 0.01 mm per 5 mm (0.2% difference) and the maximum error is 0.2 per 5 mm (4% difference). The mean differences between SlicerHeart-derived measurements and those performed on commercial 3DE platforms were insignificant for practical clinical measurements. The detailed protocol for validation of SlicerHeart 3DE import as well-quantitative comparison to commercial platforms is provided in the Appendix.

## Discussion

We present SlicerHeart, an open-source toolkit dedicated to the analysis of 3D cardiac imaging data based upon 3D Slicer. Although the initial development of these tools was focused on congenital heart disease and 3DE, SlicerHeart is relevant to many imaging modalities and patient populations. Precision medicine is a term often applied to tailoring therapy based upon genetics. However, in structural fields, personalized medicine and intervention are often predicated upon altering structure to improve function. As such, multimodality imaging forms the basis for precision medicine planning of patient specific interventions. Customization of interventions is particularly important for application to congenital heart disease, as many of the structures of interest are atypical, and it is unlikely there will be significant development of customized modeling software by industry due to the small and heterogeneous populations therein. Collaborative academic efforts thus form a critical aspect of realizing the potential for images to inform patient treatments and outcomes. In this setting the reproducible and transparent metrics are critical to the translation of innovations into improved outcomes. While these principles are clearly relevant to pediatric cardiac research, these concepts scale and translate to many areas of cardiovascular science.

For example, machine learning is a methodology which holds immense promise in cardiovascular medicine, and especially for image analysis (51). The ability to import, annotate (segment), and analyze cardiac data will be critical to create data to train machine learning networks. However, despite extensive investigation of application of machine learning to automatic segmentation and modeling of numerous organs (e.g., brain), relatively little has been published based on 3DE-based images, and in particular images of cardiac valves. We hope that the development of open-source, common platforms for the import and analysis of cardiac images, including 3DE, will accelerate the availability of annotated datasets to train networks, which in turn will allow rapid modeling, quantification, and other relevant diagnostics to be derived from clinical images (52).

Cardiovascular medicine also relies heavily on real time image-guided interventions, informed by fluoroscopy, and increasingly 3DE. However, because many of these platforms are proprietary, the possibilities for academic innovation outside of commercial development have been limited. The application of specific modules we have incorporated into SlicerHeart address some of the clinical questions we encounter in pediatric cardiology such as atrial and ventricular septal defect closure, transcatheter pulmonary valves, and baffle creation in double outlet right ventricle. Some of these tools are now used clinically at our institution, such as the baffle planner, demonstrating clinical translation (29). While our efforts have focused on application to pediatric populations, there is clear relevance of other SlicerHeart modules to larger adult populations such as those focused on planning interventions for transcatheter edge-to-edge repair, and transcatheter mitral valves (7, 23). Further, allied toolkits like the PLUS toolkit and SlicerIGT (15, 20) allow the integration of images for the active guidance of procedures themselves, as has been demonstrated in neurosurgical and abdominal procedures. With further standardization of 3DE formats and other cardiac imaging modalities, we hope similar progress can be made in cardiac medicine.

Finally, the field of cardiac mechanics also depends upon image-derived models in order to meet the promise of patient-specific computational modeling (2). SlicerHeart-derived models can be incorporated into finite-element simulations, to facilitate the creation of patient image-based simulations (53, 54). Improved access to and manipulation of image data, and, in particular, 3DE data, may help catalyze further progress in this field. To this end, we are actively working to make dedicated workflows for the creation and import of image-derived models into established open-source platforms for biomechanical modeling (53, 55, 56).

In conclusion, we hope that SlicerHeart becomes a nidus for the development of innovative applications, far beyond the original pediatric focus, and catalyzes the broad application of image-based precision medicine to cardiac disease.

## Author contributions

AL, MJ, HN, RK, SP, and SD contributed to the conception of this platform manuscript. MJ, AL, SD, GF, RK, CH, SP, SD, SS-O, and KS designed and developed the software. AL and MJ wrote the first draft of the manuscript. SD and SS-O wrote sections of the manuscript and appendix. MJ, CV, SC, and AC created figures and video content. CV, SC, and AC performed the validation of the SlicerHeart platform compared to commercial platforms. All authors contributed to manuscript revision, read, and approved the submitted version.

## Funding

This work was funded by the Department of Anesthesia and Critical Care at the Children's Hospital of Philadelphia (CHOP), a CHOP Cardiac Center Innovation Grant, the Cora Topolewski Cardiac Research Fund At CHOP, Big Hearts to Little Hearts, The Pediatric Valve Center Frontier Program at CHOP, Canarie Research Software Foundation, Ottawa, Ontario, Canada, the Natural Sciences and Engineering Research Council of Canada, Canada, and NIH R01 HL153166, P41 EB015902, P41EB028741, R01 CA235589, and the National Cancer Data Ecosystem, Task Order No. 413 HHSN26110071 under Contract No. HHSN261201500003l.

## Conflict of interest

CP is a contracted developer employed by Pixel Medical. SP is a software architect at Isomics, Inc. Both Pixel and Isomics are companies which support open-source software. The remaining authors declare that the research was conducted in the absence of any commercial or financial relationships that could be construed as a potential conflict of interest.

## Publisher's note

All claims expressed in this article are solely those of the authors and do not necessarily represent those of their affiliated organizations, or those of the publisher, the editors and the reviewers. Any product that may be evaluated in this article, or claim that may be made by its manufacturer, is not guaranteed or endorsed by the publisher.

## Supplementary material

Supplementary Figure 1 Volume ray casting. Schematic representation of volume ray casting for direct volume rendering of a 3D volume dataset. The color of each pixel is determined by casting a ray from the point of vision through the image plane, accumulating the contribution of voxels in the volume at regular intervals.

Supplementary Figure 2 Schematic of 2D view of the ATS Model 550 Phantom (CIRS Medical, Bridgeport, CT) used to validate and compare analysis performed in 3D Slicer compared to Philips QLab (Philips Medical, Andover MA) and GE Q Analysis (GE Healthcare, Chicago, IL). H1, H2 and V were the designated lines measured for the analysis. Each line is 40.0 mm consisting of nine targets each spaced 5.0 mm apart.

Supplementary Table 1 Number of Philips images and measurements.

Supplementary Table 2 Number of GE images and measurements.

Supplementary Table 3 3D echocardiography phantom validation (Philips).

Supplementary Table 4 3D echocardiography phantom validation (GE).

Supplementary Video 1 Demonstration of a workflow for importing 3D Echocardiographic image data into 3D Slicer using SlicerHeart as well as loading of tomographic images in DICOM format, and visualization of these data in 3D Slicer.

Supplementary Video 2 Demonstration of echo volume rendering module in SlicerHeart. This video also demonstrates the new rotational markups Region of Interest (ROI) functionality.

Supplementary Video 3 Mitral annular modeling and segmentation in SlicerHeart. Demonstrates a workflow for modeling an annular curve and segmenting valve leaflets of an abnormal mitral valve using modules in the SlicerHeart Extension for 3D Slicer.

Supplementary Video 4 Quantification of the mitral valve. Demonstrates a workflow for calculating annular, leaflet, and papillary muscle metrics using modules in the SlicerHeart extension for 3D Slicer.

## Abbreviations

3D: Three-dimensional
2D: Two-dimensional
3DE: 3D echocardiography/echocardiogram
API: Application Programming Interface
CAD: Computer-Aided Design
CT: Computed tomography
CMR: Cardiac magnetic resonance imaging
DICOM: Digital Imaging and Communications in Medicine
MRI: Magnetic Resonance Imaging
ROI: Region of Interest
TEE: Transesophageal Echocardiography
TTE: Transthoracic Echocardiography
VR: Virtual Reality
VTK: The Visualization Toolkit.
