# Supplementary material for: SlicerHeart: An open-source computing platform for cardiac image analysis and modeling
Source: Front Cardiovasc Med. 2022 Sep 6;9:886549. doi: 10.3389/fcvm.2022.886549 (PMC9485637; doi:10.3389/fcvm.2022.886549)
Supplement: Supplementary Figure 1 — Volume ray casting. Schematic representation of volume ray casting for direct volume rendering of a 3D volume dataset. The color of each pixel is determined by casting a ray from the point of vision through the image plane, accumulating the contribution of voxels in the volume at regular intervals. [file Data_Sheet_1.docx]

**Supplemental Material for Online Supplement**

**Appendix:**

**Part I: Technical Description of SlicerHeart Echo Volume Rendering Module**

*Volume Ray Casting*

In volume ray casting, rays are cast from a defined point of vision and go through the image plane, on which a grid of pixels is defined (Supplemental Figure S1). Rays then pass through the volume data and discrete points distributed from a distance Δs are selected along the section of the ray that passes through the volume: this is called sampling. Color and opacity values are mapped to the intensity of each sampling point. Using a process called shading, the color values are then used to determine the lighting of each sampling point. Once the shading has been computed, a method known as compositing allows to assemble the contributions from each individual sampling point. This results in a final value for the opacity and color of pixels processed in the image plane (1).

*Shading and Compositing*

The shading used in the module is performed by VTK and is based on the Phong model (2), which considers the diffuse and specular reflection of light as well as ambient lighting. In this model, the computation for the shading of a point on an object (S) can be described as shown in equation (1):

| $S = k_{a}I_{a} + \sum_{m \in lights} (k_{d}(\hat{l}\cdot\hat{n})i_{m,d} + k_{s}\left( \hat{r}\cdot\hat{v} \right)^{\alpha}i_{m,s})$ | (1) |
| --- | --- |

The term $k_{a}I_{a}$ refers to ambient lighting, where $I_{a}$ is the intensity of the ambient lighting and $k_{a}$, the ambient light coefficient. The term $k_{d}(\hat{l}\cdot\hat{n})i_{m,d}$ describes the diffuse reflection, which considers the coefficient of diffusion ($k_{d}$), the intensity of diffuse light ($i_{m,d}$), the unit vectors normal to the surface ($\hat{n}$) and the direction of the light source ($\hat{l}$). The specular reflection, measured by the term $k_{s}\left( \hat{r}\cdot\hat{v} \right)^{\alpha}i_{m,s}$, depends on the specular coefficient ($k_{s}$), the reflected ray of light ($\hat{r}$), the line of sight from the observer ($\hat{v}$), the material’s “gloss” property ($\alpha$) and the intensity of specular light ($i_{m,s}$). In the Echo Volume Render module, the normal vector $\hat{n}$ is replaced by the intensity gradient at a boundary surface.

Compositing by VTK is performed using the front-to-back alpha blending method, which can be computed as described in equation (2):

| $C_{front-back}=\sum_{i=0}^{b} \alpha_{i}C_{i}\prod_{j=1}^{i-1} (1-\alpha_{j})$ | (2) |
| --- | --- |

Where the opacity and color of each sampling point accumulated along the casted ray are represented by $\alpha_{i}$ and $C_{i}$, respectively (3).

*Depth-dependent Coloring*

In most other 3D imaging modalities, voxel intensity values convey important tissue information, such as density or water content. Therefore, image intensity is often used for coloring of the volume-rendered model. However, in cardiac 3DE, since voxel intensity is mainly utilized for extracting boundary surfaces from the image, coloring based on voxel intensity would result in homogeneous color. Depth-dependent coloring is a method currently used in commercial 3DE platforms to help clinicians interpret depth more intuitively. With this method, coloring is based on the distance of the voxel from the observer instead of depending on voxel intensity values.

We created a custom shader to implement depth-dependent coloring. This shader determines a range between the “near” (yellow) and “far” (blue) colors, which can be adjusted by the user with the “Depth range” parameter. To modulate hue and brightness depending on the distance, coloring is converted to the HSV (hue, saturation, value) model (4). Hue, saturation and brightness values are determined as follows.

| $h_{near}=h_{b}-h_{near color}$  $h_{far}=h_{b}-h_{far color}$  $s=s_{b} \times s_{s}$  $b_{near}=b_{s}\times b_{b}$  $b_{far}=b_{s}\times(b_{b}-b_{d})$ |  |
| --- | --- |

Where $h_{b}$, $s_{b}$ and $b_{b}$are the hue, saturation and brightness of the base color, while $s_{s}$, $b_{s}$ and $b_{d}$ are the user-defined values from the “depth coloring”, “saturation”, “brightness” and “depth-darkening” parameters. $h_{near color}$and $h_{far color}$are the user-defined values from the “depth coloring” parameter, which represent the offset from the base color’s hue to determine the near and far colors (which by default are yellow and blue).

HSV values are then reconverted to RGB, and coloring inside the depth range (*c*) is determined by the ratio of the distance (*d*) between “near” and “far” colors, as shown below.

| ${RGB}_{near}=hsv2rgb(h_{near}, s,b_{near})$  ${RGB}_{far}=hsv2rgb(h_{far}, s,b_{far})$  $c = (1-d)\times{RGB}_{near}+ d{\times RGB}_{far}$ |  |
| --- | --- |

*Smoothing Factor*

The “smoothing factor” in the Echo Volume Render module is a pre-processing parameter, allowing for easy application of a Gaussian smoothing filter to the 3D dataset prior to volume rendering, using a VTK implementation. In the Gaussian smoothing filter, the kernel follows a normal distribution, where the standard deviation represents the dispersion of the curve. For the “smoothing factor” parameter, the chosen value in the user interface’s slider is divided by the voxel spacing in each of the three axes, which results in the standard deviation in each dimension.

*Threshold and Edge Smoothing*

The standard volume rendering module of VTK maps voxel intensities to an opacity value using what is commonly called an opacity transfer function (OTF). This type of function can be counter-intuitive and difficult to manipulate for clinicians. The SlicerHeart Echo Volume Render module hides the complexity of manipulating a transfer function behind the “threshold” and “edge smoothing” parameters.

The “threshold” parameter shifts the opacity threshold from left to right, allowing modification of the intensity values that correspond to the upper and lower opacity limits. The value selected on the interface represents the percentage of the intensity range from 0 to 255.

The “edge smoothing” parameter stretches the opacity function horizontally to achieve a similar appearance to commercial platforms. The value chosen for edge smoothing on the interface is a percentage of the intensity range, which is then either added or subtracted from the threshold value to determine the points to the left and right side of the OTF.

**Part II: Validation of Ultrasound Derived Measurements in 3D Slicer Relative to Commercial Tools**

*Introduction*

To confirm that our implementation of both the Philips DICOM importer and the 3DImageAPI functionality was accurate, we imaged an ATS Model 550 Phantom (CIRS Inc., Norfolk VA, USA) and measured the 40mm vertical and horizontal lines with targets spaced 5mm apart (Supplemental Figure S2). We scanned using a Philips Epiq as well as GE Vivid E95 system.

*Validation Methods*

3D images were captured using the Philips X8 transesophageal (TEE), X7 transthoracic (TTE), and X5 TTE 3D probes and the GE 4V 3D TTE probe. For each set of experiments in which a parameter was varied, images were acquired with the probe position as dot to left and dot to up (90 degrees relative to one another) to verify spacing along all axes. We began by capturing a 3D image for each probe in both probe positions. Next, variable angle image series were acquired by rocking each probe to the left and to the right in both positions to acquire images with the target lines at various regions to assess accuracy throughout the ultrasound image field of view. Finally, using only the Philips X7 probe and GE 4V probe, images were captured by varying the width, elevation, and depth parameters. Four images were acquired for each parameter to ensure consistent results across a wide range of acquisition parameters. In total, 24 images were acquired for Philips validation and 12 images were acquired for GE validation. Supplemental Table S1 and Supplemental Table S2 provide a distribution of the number of images and measurements for each platform.

The next step of the validation protocol was to measure the standardized vertical and horizontal line targets using the distance measurement tools in Philips QLab, GE Q Analysis, and 3D Slicer to confirm accuracy of image reconstruction in 3D Slicer and commercial platforms. Philips images were imported into Philips QLab (Philips Medical, Andover, MA, USA), saved in DICOM format, and imported into 3D Slicer. GE images were analyzed using GE Q Analysis and then imported into 3D Slicer using the Image3dAPI. Measurements for Philips were completed by two operators and measurements for GE by one operator. Each operator carefully drew a line between the centers of the two outermost, fully visible targets for lines H1, H2, and V resulting in six individual measurements (three from both platforms) for each image. In total, each observer completed 90 individual line measurements in 3D Slicer and Philips QLab and 39 individual line measurements in 3D Slicer and GE Q Analysis. In most images, eight 5mm segments were visible (40mm total) but in other images, the entire 40mm length was only partly visible. This frequently occurred during the variable angle image series where the probe was rocked to create a series of images with the target lines at the edge of the ultrasound image field of view. Therefore, we normalized the measurements to one 5mm segment to ensure comparable measurements.

*Results*

Supplemental Table S3 and Supplemental Table S4 demonstrate a comparison of the measurement values between 3D Slicer and Philips QLab and 3D Slicer and GE Q Analysis. Measurements in 3D Slicer were comparable to measurements in Philips QLab and GE Q Analysis. In comparing measurements between Philips QLab and 3D Slicer, the mean difference is 0.04mm per 5mm (0.8% difference) with a maximum error of 0.1mm per 5mm (2% difference). In comparing measurements between GE Q Analysis and 3D Slicer, the mean difference is 0.01mm per 5mm (0.2% difference) and the maximum error is 0.2 per 5mm (4% difference). Of note, on the GE platform, measurements are limited to whole millimeters and as such, this difference in measurement precision could have led to a greater maximum error for GE Q Analysis compared to 3D Slicer or Philips QLab. All of these differences are small and are in the range of variation of human measurement.

**Appendix Figures and Legends
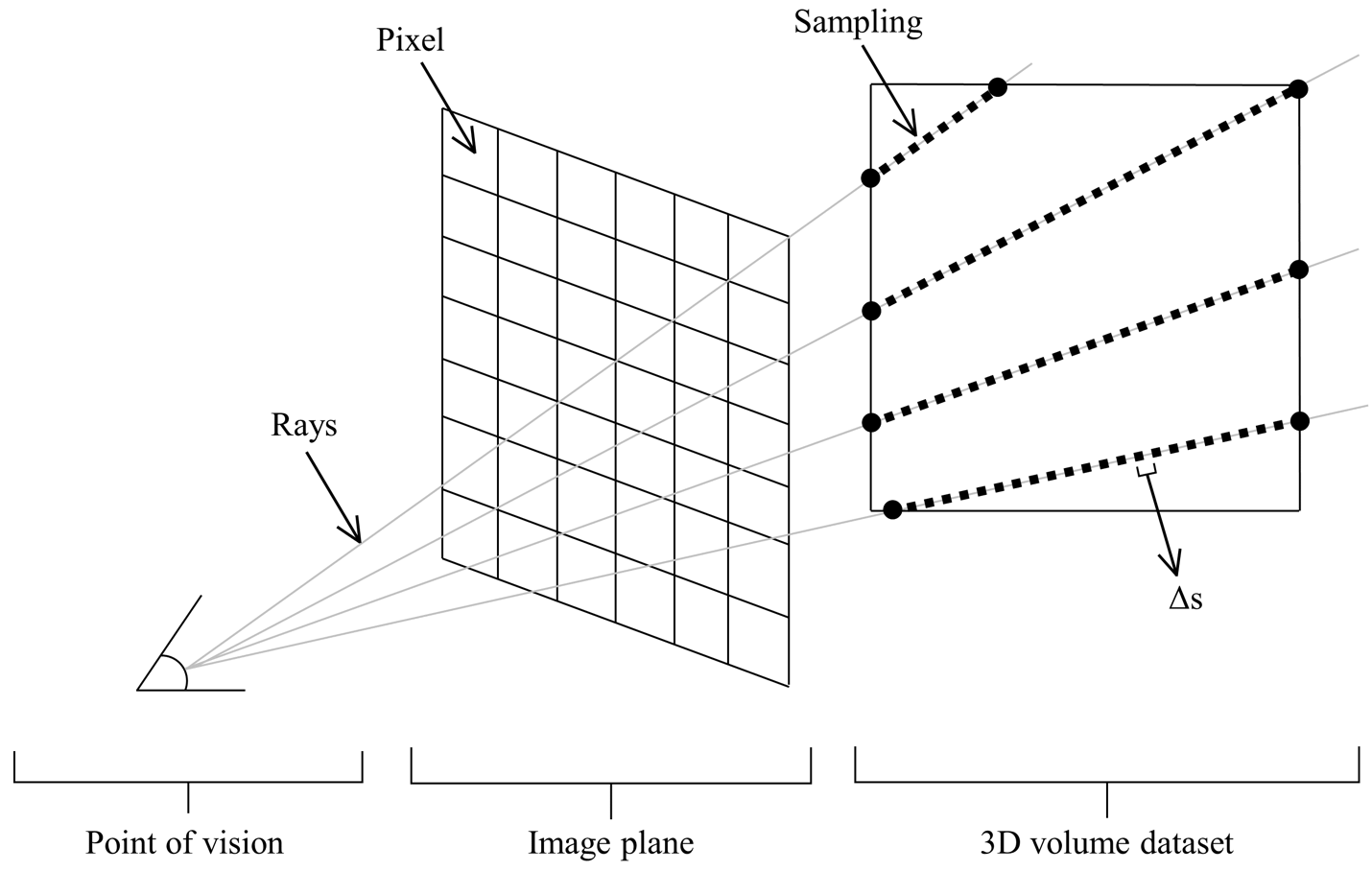
**

**Supplemental Figure S1. Volume Ray Casting.** Schematic representation of volume ray casting for direct volume rendering of a 3D volume dataset. The color of each pixel is determined by casting a ray from the point of vision through the image plane, accumulating the contribution of voxels in the volume at regular intervals.


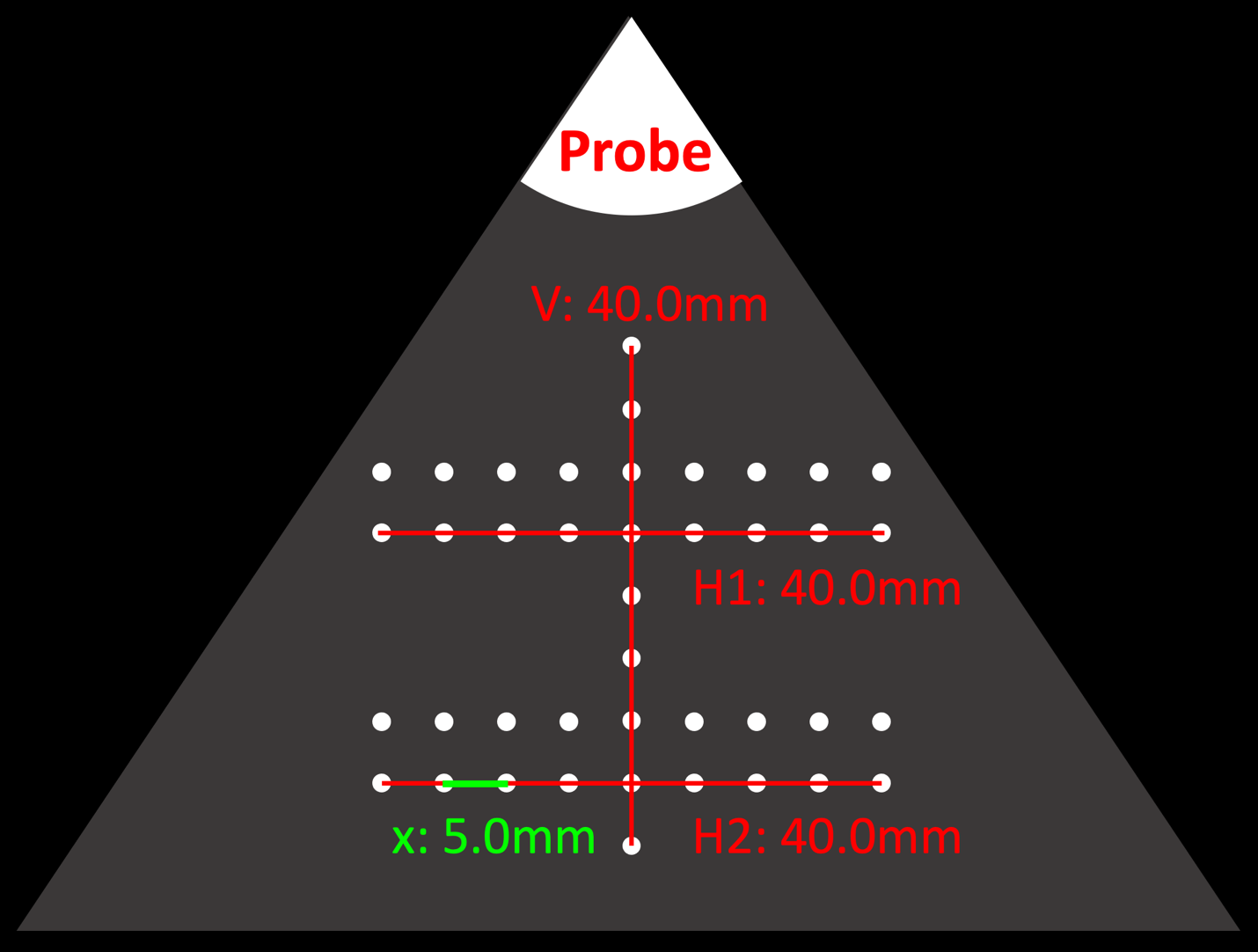


**Supplemental Figure S2.** Schematic of 2D view of the ATS Model 550 Phantom (CIRS Medical, Bridgeport, CT) used to validate and compare analysis performed in 3D Slicer compared to Philips QLab (Philips Medical, Andover MA) and GE Q Analysis (GE Healthcare, Chicago, IL). H1, H2 and V were the designated lines measured for the analysis. Each line is 40.0 mm consisting of nine targets each spaced 5.0mm apart.

**Appendix Tables**

| ***Supplementary Table S1. Number of Philips Images and Measurements*** | | | | |
| --- | --- | --- | --- | --- |
| **Parameter** | **Dot Position** | **Probe** | **Images** | **Measurements** |
| 3D Still Image  Image Series (Right to Left) | Left and Up  Left and Up | X8, X7, X5  X8, X7, X5 | 6  6 | 18  36 |
| Varied Width | Left | X7 | 4 | 12 |
| Varied Depth | Left | X7 | 4 | 12 |
| Varied Elevation | Left | X7 | 4 | 12 |
| **Total** |  |  | **24** | **90** |

| ***Supplementary Table S2. Number of GE Images and Measurements*** | | | | |
| --- | --- | --- | --- | --- |
| **Parameter** | **Dot Position** | **Probe** | **Images** | **Measurements** |
| 3D Still Image  Image Series (Right to Left) | Left and Up  Left and Up | 4V  4V | 2  2 | 6  9 |
| Varied Width | Left | 4V | 4 | 12 |
| Varied Depth | Left | 4V | 4 | 12 |
| **Total** |  |  | **12** | **39** |

| ***Supplementary Table S3. 3D Echocardiography Phantom Validation (Philips)*** | | | |  |
| --- | --- | --- | --- | --- |
| **Normalized Measurements by Probe** | **n** | **3D Slicer (mm)** | **Philips QLab (mm)** | |
| **Overall**  **Overall X5** | 90  18 | 5.0 [5.0-5.1]  5.0 [5.0-5.1] | 5.0 [5.0-5.0]  5.0 [4.9-5.1] | |
| H1 | 6 | 5.1 [5.0-5.1] | 5.0 [4.9-5.0] | |
| H2 | 6 | 5.0 [5.0-5.1] | 4.9 [4.9-5.0] | |
| V | 6 | 5.1 [5.0-5.1] | 5.0 [5.0-5.1] | |
| **Overall X7** | 54 | 5.1 [5.0-5.3] | 5.1 [5.0-5.3] | |
| H1 | 18 | 5.3 [5.2-5.3] | 5.2 [5.2-5.3] | |
| H2 | 18 | 5.1 [5.1-5.2] | 5.1 [5.0-5.1] | |
| V | 18 | 5.0 [5.0-5.1] | 5.0 [5.0-5.0] | |
| **Overall X8** | 18 | 5.1 [5.0-5.2] | 5.1 [4.9-5.1] | |
| H1 | 6 | 5.2 [5.1-5.2] | 5.1 [5.1-5.1] | |
| H2 | 6 | 5.1 [5.0-5.2] | 5.1 [5.0-5.1] | |
| V | 6 | 5.0 [4.9-5.0] | 5.0 [4.9-5.0] | |

| ***Supplementary Table S4. 3D Echocardiography Phantom Validation (GE)*** | | | |  |
| --- | --- | --- | --- | --- |
| **Normalized Measurements (mm)** | **n** | **3D Slicer (mm)** | **GE Q Analysis (mm)** | |
| **Overall** | 39 | 5.1 [5.0-5.2] | 5.0 [5.0-5.3] | |
| H1 | 13 | 5.2 [5.1-5.3] | 5.3 [5.0-5.3] | |
| H2 | 13 | 5.1 [5.0-5.1] | 5.0 [5.0-5.3] | |
| V | 13 | 5.1 [5.0-5.1] | 5.0 [5.0-5.0] | |

**References**

1. Preim B, Botha C. Chapter 7 - Direct Volume Visualization. In: Preim B, Botha C, editors. Visual Computing for Medicine (Second Edition). Boston: Morgan Kaufmann, 2014:269-287.

2. Phong BT. Illumination for computer generated pictures. Commun ACM 1975;18:311–317.

3. Bozorgi M, Lindseth F. GPU-based multi-volume ray casting within VTK for medical applications. Int J Comput Assist Radiol Surg 2015;10:293-300.

4. Smith AR. Color gamut transform pairs. Proceedings of the 5th annual conference on Computer graphics and interactive techniques: Association for Computing Machinery, 1978:12–19.
